# Recent Increase in Meningitis Caused by *Neisseria meningitidis* Serogroups A and W135, Yaoundé, Cameroon

**DOI:** 10.3201/eid0803.010308

**Published:** 2002-03

**Authors:** Marie-Christine Fonkoua, Muhamed-Kheir Taha, Pierre Nicolas, Patrick Cunin, Jean-Michel Alonso, Raymond Bercion, Jeanne Musi, Paul M.V. Martin

**Affiliations:** * Centre Pasteur du Cameroun, Yaoundé, Cameroun; † Institut Pasteur, Paris, France; ‡ Centre Collaborateur de l’OMS, Institut de Médecine Tropicale du Service de Santé des Armées, Marseille, France

**Keywords:** *Neisseria meningitidis*, serogroups, W135, Africa

## Abstract

From 1991 to 1998, *Neisseria meningitidis* serogroups A, B, and C represented 2%-10% of strains isolated from cases of bacterial meningitis in Yaoundé. During 1999 to 2000, the percentage of meningococci reached 17%, a proportion never reported since recordkeeping began in 1984. The increase of serogroup A meningococci and the emergence of W135 strains highlight the need for increased surveillance for better diagnosis and prevention.

*Neisseria meningitidis* serogroup A causes major epidemics of meningitis in Africa, essentially within the African meningitis belt (*1*)**.** Epidemics of cerebrospinal meningitis in this belt are often enormous (*1*). During the first 9 months of 1996 in the World Health Organization (WHO) African Region, 146,166 cases were reported to WHO; 15,783 were fatal. During that year, 42,129 cases occurred in Burkina Faso, 7,244 in Mali, 16,050 in Niger, and 75,069 in Nigeria. These four countries reported 95% of the cases in Africa in 1996, for an overall case-fatality rate of 10.6% (*2*).

The recommended control practices in Africa involve vaccination with the meningococcal bivalent polysaccharide A/C vaccine in response to epidemics. Efficient public health practice necessitates that epidemics be detected early, stocks of vaccines be set up in target regions, and field vaccination with the bivalent vaccine be rapid, since the quadrivalent ACYW135 vaccine has limited worldwide supply and is more expensive.

The presence of *N. meningitidis* serogroup W135 has been confirmed in Africa for some time. In Burkina Faso in 1980, 1.3% of the meningococcal strains isolated from rhinopharyngeal carriers belonged to serogroup W135. In 1981 and 1982, monitoring of the serogroups responsible for meningococcal meningitis at Dakar (Senegal) and Niamey (Niger) showed that 4% and 3% of strains, respectively, belonged to serogroup W135 (*3*). In 1984 and 1985**,** 7% of *N. meningitidis* strains isolated from meningitis cases in Gambia belonged to serogroup W135 (*4*). In 1993 and 1994, two strains of *N. meningitidis* W135 were isolated from patients in Mali; both belonged to the ET-37 complex (*5*). More recently, in 1994, six strains of serogroup W135 isolated from clinical cases in Gambia were studied; they also belonged to the ET-37 complex. DNA macrorestriction analysis of these strains identified four different profiles in pulsed-field gel electrophoresis (PFGE), indicating that the strains involved were closely related but different (*6*). W135 strains are often isolated after intensive campaigns of vaccination against meningococci of serogroups A and C (*3*,*4*,*6*).

In spring 2000, an epidemic of *N. meningitidis* W135 infection broke out among Hajj pilgrims (for whom vaccination against meningococci of serogroups A and C is mandatory) and their close contacts. In all, 241 cases were reported in Saudi Arabia and 90 in 13 other countries (*7*), including the United States (4 cases) (*8*), the United Kingdom (33 cases), and France (19 cases). All these strains showed markers of the ET-37 complex; had an antigenic formula W135:2a:P1-5,2; a sequence type ST-11; and the same profile on PFGE (*9*), confirming the clonal origin of the epidemic. Four W135 strains isolated in U.S. patients epidemiologically linked to Hajj pilgrims were further studied. The sequence of the *porA* gene showed that these four strains had variable regions VR1 and VR2 identical to those of the prototype P1.5,2 strain (*8*).

Apparently, the W135 strains isolated in Africa until 1995 did not cause large epidemics, even if isolated in the countries in the African meningitis belt in which epidemics due to serogroups A meningococci are frequent (e.g., Niger, Mali, Senegal, and Gambia). In Niger in 1981, only one W135 strain of 231 meningococci was isolated from a meningitis case (*3*). Similarly, W135 accounted for 7 of 42 strains in 1982 in Niger, and 3 of 76 strains in Senegal in 1981 to 1982 (*3*), 3 of 41 in Guinea in 1984 to 1985 (*4*), and 2 of 75 strains isolated in 1991 to 1994 in six countries in the African meningitis belt (*5*). However, available information shows that the case-fatality rate due to W135 strains was relatively high in Africa before 1995, as in Europe during the recent Hajj 2000 epidemic: 6 (35%) of 17 cases in Africa before 1995 (in Senegal, Gambia, and Niger) and 10 (18%) of 56 cases in Europe in 2000 (in the United Kingdom, France, and the Netherlands).

## The Study

We report here a sudden increase in the number of meningococcal strains isolated from cerebrospinal fluid (CSF) sent to the Medical Biology Laboratory of the Pasteur Centre of Cameroon (CPC) at Yaoundé in the 1-year period 1999 to 2000 (note that in Cameroon the administrative year begins on July 1). Yaoundé, the capital of Cameroon, is a city of approximately 1,500,000 inhabitants. Located in the forest zone at an altitude of 750 m, about 400 km south of the southern limit of the African meningitis belt, it has a humid, tropical climate. The CPC laboratory receives samples from patients admitted to the principal hospitals of Yaounde and, in 1999 to 2000**,** 91.5% of CSF samples sent to the laboratory were from children <15 years old; 81% were from children <5 years.

From 1984 to 1990, bacteria were isolated from 767 (5.8%) of 13,134 CSF samples; 42 (5.5%) of these were *N. meningitidis.* This proportion was significantly lower than during the 1991 to 2000 period (8.7%; p<0.05). We do not know if there were changes in the population of patients using the CPC services that might account for the slow increase in cases of meningococcal disease. Slow improvement of laboratory practices and medical competence might account for slowly increasing proportions of *N. meningitidis* over a 20-year period. However, no major changes in laboratory techniques occurred in 1999 at CPC that could account for the sudden increase observed in 1999 and 2000. Moreover, during that 20-year period**,** most CSF samples came from the same pediatric wards of the neighboring Central Hospital of Yaoundé and other major children’s hospitals.

The table shows the changes in isolation rates of *N. meningitidis* from clinical cases at Yaoundé during 1991-92 to 2000-01. The number of meningococcal strains isolated has remained small for the last 10 years, as would be expected in a zone located at a considerable distance south of the African meningitis belt and one in which pneumococci and *Haemophilus influenzae* are the two most frequent bacterial agents of meningitis. Most meningococcal strains isolated were serogroup A, the most frequent group in Africa. The proportion of meningococci identified in cases of bacterial meningitis varied significantly in this period (p<0.01).

**Table:** Isolation of *Neisseria meningitidis* from meningitis cases at Yaoundé, Cameroon, 1991–2001

Year	No. of CSF samples	No. (%) of cases of bacterial meningitis	No. (%) of cases of meningococcal meningitis	No. of strains of each serogroup
1991-1992	1,246	131(10.5)	8(6.1)	6 A; 2 C
1992-1993	1,049	105(10)	11(10.5)	11 A
1993-1994	961	88(9.2)	9(10.2)	8 A; 1 B
1994-1995	722	69(9.6)	6(8.7)	2 A; 4B
1995-1996	998	70(7)	4(5.7)	1A; 1B; 1C; 1NT*
1996-1997	1,255	97(7.7)	2(2.1)	2C
1997-1998	1,282	92(7.2)	6(6.5)	4A; 1C; 1NT
1998-1999	1,505	116(7.7)	8(6.9)	6A; 1B; 1W135
1999-2000	1,812	120(6.6)	23(19.2)	17A; 2B; 4W135
2000-2001	1,612	81(5)	17(21)	13A; 4W135
Total	1,2442	969 (7.8)	94 (9.7)	68A; 9B; 6C;9W135; 2NT

NT: not serogrouped; CSF: cerebrospinal fluid.

In the 2-year period 1998 to 2000**,** two events occurred. The first was an increase in the number of isolates of *N. meningitidis*; such isolates accounted for >19% of strains isolated from cases of bacterial meningitis in 1999 to 2000, and 21% in 2000 to 2001—two to three times more than normal. We checked records back to 1984 and found that in none of the years in this period was such a large number of meningococci isolated at Yaoundé. The second noteworthy event was the appearance of serogroup W135 strains, which accounted for 9 (19%) of 48 meningococcal strains isolated in 1998 to 2000 versus 0 of 46 in 1991 to 1997 (p<0.01).

One W135 case occurred in January 1999 in a 12-year-old boy from Yaoundé who had no known contact with a Hajj pilgrim and no recent history of travel. On the four W135 patients from 1999 to 2000, one was male and three were female. Ages were 2, 3, 29, and 37 years. Onsets of disease were in July 1999 and in May and June 2000, i.e., after the usual meningococcal peak in the dry season, and none had known direct or indirect contact with each other or with a Hajj pilgrim. In 2000 to 2001, four cases occurred, all in males (aged 9, 15, 23, and 40 years); onsets of diseases occurred in January 2001, then in March, May, and June. One of them was in a 23-year-old student, who had been studying in Dakar (Senegal) for 2 years; he became ill while observing holy days in Cameroon. He could have been in indirect contact with Hajj pilgrims, since he was Muslim and Senegal is largely Muslim. Vaccination status was obtained for five of these nine patients: two were vaccinated against meningococcal meningitis, including the student.

The five strains of *N. meningitidis* W135 isolated in 1999 to 2000 were serotyped, subtyped, and studied by molecular biology techniques. All belonged to the ET-37 complex and had the antigenic formula W135:2a:P1.2,5. Two strains were subjected to multilocus sequence typing; both were of sequence type ST-11, typical of isolates of the ET-37 complex (*10*). These five strains were indistinguishable by multilocus DNA fingerprinting and showed markers of E-37 complex (*11*). Finally, *Spe*I restriction profiles were determined by PFGE: four of the strains were indistinguishable, and the final strain differed by one band only. All these clones differed slightly (by two bands for four isolates and by three bands for one isolate) from the clone isolated from the Hajj pilgrims in 2000.

## Conclusions

These results show an increase of serogroup A meningococci in Yaoundé and demonstrate the presence and circulation of at least one indigenous clone of *N. meningitidis* W135 of the ET-37 complex in Central Africa. The clone is very similar to, but differs slightly from, the clone responsible for a meningitis outbreak among Hajj pilgrims in 2000 (*8*,*9*). Since none of the patients with W135 meningococci had direct contact with Hajj pilgrims and Cameroonian W135 strains are slightly different by PFGE from the W135 clone isolated in Europe and the United States in 2000, these strains from Cameroon seem to predate the 2000 Hajj-associated outbreak. A larger study of the W135 strains isolated in Africa, Europe, and Asia, from patients with no direct link to the pilgrimage to Mecca (indigenous strains) would make it possible to identify the geographic origin of the strain responsible for the Mecca epidemic in 2000. Such studies would also make it possible to elucidate the role of A and C vaccination in the selection of W135 clones belonging to the ET-37 complex.

 We cannot explain with certainty why serogroup A meningococci has increased in Yaoundé, but the finding stresses the importance of continuous surveillance. The circulation of W135 strains in Central Africa raises questions about their epidemic potential and highlights the microbiologic surveillance of meningococcal meningitis. Thus, anti-W135 serogrouping antibodies are necessary for all National Reference Laboratory services. Antigen-detection kits for the diagnosis of meningitis should also contain anti-W135 antibodies. Moreover, the problem of the availability of a quadrivalent vaccine, including the W135 antigen, should be resolved. Strengthening the capacities for epidemiologic and microbiologic surveillance of meningitis in Africa is a prerequisite for prevention and control of meningococcal epidemics.
